# Effect of dexmedetomidine versus lorazepam on outcome in patients with sepsis: an *a priori*-designed analysis of the MENDS randomized controlled trial

**DOI:** 10.1186/cc8916

**Published:** 2010-03-16

**Authors:** Pratik P Pandharipande, Robert D Sanders, Timothy D Girard, Stuart McGrane, Jennifer L Thompson, Ayumi K Shintani, Daniel L Herr, Mervyn Maze, E Wesley Ely

**Affiliations:** 1 Anesthesiology Service, VA TN Valley Health Care System, 1310 24th Avenue South, Nashville, TN 37212-2637, USA; 2 Department of Anesthesiology, Division of Critical Care, Vanderbilt University School of Medicine; 324 MAB, Nashville, TN 37212-1120, USA; 3 Department of Leucocyte Biology & Magill Department of Anaesthetics, Intensive Care and Pain Medicine, Imperial College London, Chelsea & Westminster Hospital, 369 Fulham Road, London, SW10 9NH, UK; 4 Department of Medicine, Division of Allergy, Pulmonary, and Critical Care Medicine, Vanderbilt University School of Medicine; T-1218 MCN, Nashville, TN 37232-2650, USA; 5 Center for Health Services Research, Vanderbilt University School of Medicine; 6th Floor MCE, Suite 6100, Nashville, TN 37232-8300, USA; 6 Veterans Affairs Tennessee Valley Geriatric Research, Education, and Clinical Center; 1310 24th Avenue South, Nashville, TN 37212-2637, USA; 7 Department of Biostatistics, Vanderbilt University School of Medicine; S-2323 MCN, Nashville, TN 37232-2158, USA; 8 Department of Surgery and Surgical Critical Care, Washington Hospital Center; 110 Irving St NW, Room 4B42, Washington, DC 20010, USA; 9 Department of Anesthesiology and Perioperative Care, University of California San Francisco; 521 Parnassus Avenue, C455, San Francisco, CA 94143-0648, USA

## Abstract

**Introduction:**

Benzodiazepines and α_2 _adrenoceptor agonists exert opposing effects on innate immunity and mortality in animal models of infection. We hypothesized that sedation with dexmedetomidine (an α_2 _adrenoceptor agonist), as compared with lorazepam (a benzodiazepine), would provide greater improvements in clinical outcomes among septic patients than among non-septic patients.

**Methods:**

In this *a priori*-determined subgroup analysis of septic vs non-septic patients from the MENDS double-blind randomized controlled trial, adult medical/surgical mechanically ventilated patients were randomized to receive dexmedetomidine-based or lorazepam-based sedation for up to 5 days. Delirium and other clinical outcomes were analyzed comparing sedation groups, adjusting for clinically relevant covariates as well as assessing interactions between sedation group and sepsis.

**Results:**

Of the 103 patients randomized, 63 (31 dexmedetomidine; 32 lorazepam) were admitted with sepsis and 40 (21 dexmedetomidine; 19 lorazepam) without sepsis. Baseline characteristics were similar between treatment groups for both septic and non-septic patients. Compared with septic patients who received lorazepam, the dexmedetomidine septic patients had 3.2 more delirium/coma-free days (DCFD) on average (95% CI for difference, 1.1 to 4.9), 1.5 (-0.1, 2.8) more delirium-free days (DFD) and 6 (0.3, 11.1) more ventilator-free days (VFD). The beneficial effects of dexmedetomidine were more pronounced in septic patients than in non-septic patients for both DCFDs and VFDs (P-value for interaction = 0.09 and 0.02 respectively). Additionally, sedation with dexmedetomidine, compared with lorazepam, reduced the daily risk of delirium [OR, CI 0.3 (0.1, 0.7)] in both septic and non-septic patients (P-value for interaction = 0.94). Risk of dying at 28 days was reduced by 70% [hazard ratio 0.3 (0.1, 0.9)] in dexmedetomidine patients with sepsis as compared to the lorazepam patients; this reduction in death was not seen in non-septic patients (P-value for interaction = 0.11).

**Conclusions:**

In this subgroup analysis, septic patients receiving dexmedetomidine had more days free of brain dysfunction and mechanical ventilation and were less likely to die than those that received a lorazepam-based sedation regimen. These results were more pronounced in septic patients than in non-septic patients. Prospective clinical studies and further preclinical mechanistic studies are needed to confirm these results.

**Trial Registration:**

NCT00095251.

## Introduction

Recent advances in critical care medicine have identified acute brain dysfunction (delirium and coma) as a highly prevalent manifestation of organ failure in critically ill patients that is associated with increased morbidity and mortality [1-6]. Accumulating evidence also shows that the degree [7] and duration [3,8] of acute brain dysfunction are important risk factors for adverse clinical outcomes. The presence of delirium and coma can potentially worsen outcomes in septic patients [9-11]; this may be linked to septic perturbation of inflammatory, coagulopathic and neurochemical mechanisms that can contribute to the pathogenesis of acute brain dysfunction [12,13].

Sedative and analgesic medications, routinely administered to mechanically ventilated (MV) patients [14], contribute to increased time on MV and ICU length of stay [15]. Benzodiazepines, in particular, enhance the risk of developing acute brain dysfunction [6,16-18]. Other studies have demonstrated that benzodiazepines are associated with worse clinical outcomes when compared with either propofol or with opioid-based sedation regimens [19,20], although these studies did not evaluate the role of changing sedation paradigms on acute brain dysfunction.

The Maximizing Efficacy of Targeted Sedation and Reducing Neurological Dysfunction (MENDS) trial [21] demonstrated that dexmedetomidine (DEX) [22], an alpha_2 _(α_2_) adrenoceptor agonist, provided safe and efficacious sedation in critically ill MV patients, with significant improvement in brain organ dysfunction (delirium and coma) compared with the benzodiazepine, lorazepam (LZ). The principal findings from the MENDS trial were recently corroborated by the Safety and Efficacy of Dexmedetomidine Compared With Midazolam (SEDCOM) trial of 366 critically ill patients, which showed a reduction in the prevalence of delirium in patients sedated with DEX compared with midazolam; patients on DEX also showed a reduction in the duration of MV [23]. In the absence of knowledge of the mechanisms whereby DEX improves patient outcome, it will be necessary to postulate testable hypotheses; hypothesis-testing data can provide the basis for designing future comparative efficacy trials for sedation for the wide-range of ICU patients.

The α_2 _adrenoceptor agonists and benzodiazepines have different molecular targets (α_2 _adrenoceptors and gamma-aminobutyric acid type A (GABA_A_) receptors, respectively) and neural substrates for their hypnotic effects that may play a critical role in maintaining sleep architecture in critically ill patients [22,24]; improved sleep may potentially improve delirium outcomes and immune function [25-27]. In addition, benzodiazepines and α_2 _adrenoceptor agonists exert opposing effects on innate immunity, apoptotic injury and mortality in preclinical models of infection [27]. Benzodiazepines increase mortality in animal models of bacterial infection [28-30] likely by impairment of neutrophil [31] and macrophage function [32], whereas GABA_A _receptor antagonists are under investigation as anti-infective agents [33]. Contrastingly, α_2 _adrenoceptor agonists enhance macrophage phagocytosis and bacterial clearance [34-36], while exerting minimal effect on neutrophil function [37], and are associated with improved outcomes in animal models of bacterial sepsis [38]. DEX *per se *exerts superior anti-inflammatory and organ-protective properties compared with other sedatives [22,39,40] and is neuroprotective in models of hypoxia-ischemia [41] and apoptosis [42], and thus may prevent sepsis-induced brain and other organ injury. The anti-apoptotic effects of DEX are greater than midazolam [40,42] and may be useful, given that sepsis-related mortality has been associated with apoptotic injury [43]. Sympatholysis has also been shown to improve outcome in sepsis [44]; in line with previous reports [22], presumptive evidence for the more profound sympatholytic actions of DEX over its benzodiazepine comparators was suggested by the higher incidence of bradycardia and reduced tachycardia in both the MENDS [21] and SEDCOM [23] studies.

Multiple levels of evidence thus converge to support our hypothesis that sedation with DEX may lead to better outcomes for patients with sepsis than benzodiazepine sedation. We therefore conducted an *a priori*-planned subgroup analysis among patients from the MENDS trial to determine if sedation with DEX compared with LZ in septic and non-septic patients affected clinical outcomes, including duration and prevalence of acute brain dysfunction and 28-day mortality.

## Materials and methods

The MENDS study (Trial Registration Identifier: NCT00095251), conducted between August 2004 and May 2006, [21] was approved by the institutional review boards at Vanderbilt University Medical Center and Washington Hospital Center. After obtaining informed consent from either the patient or an approved surrogate, patients were randomized in a double-blind fashion to receive DEX-based (maximum 1.5 mcg/kg/hr) or LZ-based (maximum 10 mg/hr) sedation for up to five days, titrated to target Richmond Agitation-Sedation Scale (RASS) [45,46] scores determined by the managing ICU team each day. Patients were monitored daily for delirium with the Confusion Assessment Method for the ICU [1,47]. A detailed study protocol has been previously described [21]. In this subgroup analysis, we compared the effects of DEX and LZ in patients with sepsis with the effects of these sedatives in patients without sepsis. Patients were classified as being septic if they had at least two systemic inflammatory response syndrome (SIRS) criteria and a known or suspected infection between admission to within 48 hours of enrollment. A patient was 'suspected' to have an infection if the treating physicians stated this in the medical record or started antibiotics or drotrecogin alfa (activated). SIRS criteria and known/suspected infection were recorded by study personnel prospectively, and one author (TG), blinded to study group assignment, also confirmed each case of sepsis by retrospectively examining electronic medical records. Apart from sedation, all other aspects of medical management were according to standardized ventilator management protocols and sepsis treatment algorithms, provided by the critical care team, blinded to the sedative intervention.

### Primary and secondary outcomes

The primary outcome of interest was delirium/coma-free days, defined as the days alive without delirium or coma during the 12-day study period [21]. Secondary outcomes of the study included delirium-free days, daily prevalence of delirium while patients received study drug, coma-free days, lengths of stay on the MV and in the ICU, and 28-day mortality. Ventilator-free days were calculated as the number of days alive and off MV over a 28-day period [48].

Delirium was measured daily until hospital discharge or for 12 days using the Confusion Assessment Method for the ICU (CAM-ICU) [1,47]. Efficacy of the study drug was defined as the ability to achieve a sedation score within one point of the desired goal sedation level determined by the managing ICU team each day. Sedation level was assessed using the RASS [45,46], a highly reliable and well-validated sedation scale for use within patients over time in the ICU. Both the RASS and the CAM-ICU instruments are described in more detail at [49].

For other outcomes, patients were followed in the hospital from enrollment for 28 days, or until discharge or death if earlier.

### Statistical analysis

Data were analyzed using an intention-to-treat approach. Continuous data were described using medians and interquartile ranges or means and standard deviations, and categorical data using frequencies and proportions. We used Pearson chi-squared tests for categorical variables and Wilcoxon rank-sum tests for continuous variables to test for baseline differences between the two study groups, stratifying by the presence or absence of sepsis.

We used multivariable regression to examine associations between treatment group and outcomes, assessing for interactions between sepsis and the effect of treatment group on each outcome (i.e., testing for homogeneity of treatment effect according to presence or absence of sepsis). All regression models included sepsis, treatment group, and a treatment group by sepsis interaction term as independent variables, in addition to the following covariates: age, severity of illness according to the acute physiology component of the Acute Physiology and Chronic Health Evaluation (APACHE) II score at enrollment, and use of drotrecogin alfa (activated) within 48 hours of enrollment. Because the trial was not powered to detect interactions, we considered an interaction term *P *value of less than 0.15 to be significant, indicating that the treatment group affected the outcome in question differently among septic and non-septic patients.

For the primary outcome, we used bootstrap multiple linear regression to calculate a non-parametric 95% confidence interval (CI) for the adjusted difference in mean delirium/coma-free days between the two treatment groups, because of the skewed distribution of this outcome variable. Specifically, we fitted a multiple linear regression model (which included the independent variables described above) in each of 2,000 datasets randomly generated from the original data using the bootstrap method (i.e., resampling with replacement) and determined the 95% CI of the adjusted difference in mean delirium/coma-free days using the 2.5 and 97.5 percentiles of the 2,000 regression coefficients of these models. The same approach was used to analyze delirium-free days, coma-free days, and ventilator-free days.

For time-to-event outcomes (time to ICU discharge and death), Cox proportional hazards models were used. Kaplan-Meier survival curves were created for graphical representation of these time-to-event outcomes. When examining 28-day mortality, patients were censored at the time of last contact alive or at 28 days from enrollment, whichever was first. Censoring for ICU or hospital discharge analyses occurred at time of death or, rarely, at study withdrawal.

To examine the effect of treatment group on the probability of being delirious each day during the study drug period (compared with having a normal mental status), we used Markov logistic regression. These models, with an outcome of daily mental status, adjust for the previous day's mental status as well as the relevant covariates described above. Due to the multiple assessments included for each patient, generalized estimating equations were applied to this regression model to account for the correlation of these observations within each patient.

For all results except for interaction terms, two-sided *P *values of 0.05 or less were considered to indicate statistical significance. We used R (version 2.10) for all statistical analyses.

## Results

### Demographics

Sixty-three patients in the MENDS study [21] met the consensus criteria definition of sepsis, with 31 randomized to receive DEX and 32 randomized to receive LZ. Forty patients without sepsis were enrolled, of which 21 were randomized to the DEX group and 19 to the LZ group. Baseline demographics and clinical characteristics according to treatment group and sepsis are shown in Table 1. Among non-septic patients, many were admitted with pulmonary diseases, including: pulmonary embolus, pulmonary hypertension, and pulmonary fibrosis (n = 13); acute respiratory distress syndrome without infections (n = 3); and chronic obstructive pulmonary disease (n = 2). Other admission diagnoses among non-septic patients included cardiac surgery (n = 6); malignancies (n = 3), airway obstruction (n = 2); hemorrhagic shock (n = 2); gastrointestinal surgery (n = 2); neuromuscular disease (n = 1); coagulopathy (n = 1) and other surgeries (n = 5). Sepsis management was similar between septic patients receiving DEX and LZ with regard to number of antibiotics (2 (1, 3) vs 2 (1, 3), *P *= 0.37), percentage of patients receiving antibiotics on study day 1 (81% vs 81%, *P *= 0.94), and percentage treated with corticosteroids (61% vs 59%, *P *= 0.90). Although not statistically significant, drotrecogin alfa (activated) administration may have been less common among DEX septic patients than LZ septic patients (21% vs 35%, *P *= 0.20) despite a similar severity of illness according to APACHE II scores (Table 1).

**Table 1:** Baseline characteristics of patients with and without sepsis

	Patients with sepsis		Patients without sepsis	
		
Variable	DEX (n = 31)	LZ (n = 32)	DEX (n = 20)	LZ (n = 19)
Age	60 (46 to 65)	58 (44 to 66)	61 (50 to 68)	60 (52 to 67)
Males	58%	41%	57%	53%
APACHE II	30 (26 to 34)	29 (24 to 32)	27 (20 to 31)	25 (20 to 30)
SOFA score	10 (9 to13)	9 (8 to 12)	9 (8 to 12)	8 (7 to 9)
IQCODE at enrollment	3 (3 to 3)	3 (3 to 3)	3 (3 to 3)	3 (3 to 3)
Medical ICU	77%	81%	62%	47%
Surgical ICU	23%	19%	38%	53%
Pre-enrollment lorazepam (mg)	1.5 (0 to 5)	0 (0 to 4)	0 (0 to 4)	0 (0 to 2)
Enrollment RASS	-3 (-4 to -2)	-4 (-4 to -3)	-3 (-4 to 0)	-3 (-4 to -1)
**SIRS criteria**				
Temperature (Fahrenheit)	37.5 (37 to 38.3)	38 (37.2 to 38.6)	36.7 (35.8 to 37.8)	37.2 (36.2 to 38.3)
White blood count (10^3^/μL)	12.5 (6.6 to 21.7)	12.5 (7.7 to 18.8)	14.6 (8.9 to17.9)	10 (7.5 to14)
Systolic BP (mm Hg)	88 (78 to 100)	83 (79 to 100)	92 (90 to 100)	90 (80 to110)
Heart rate (per minute)	113 (100 to 134)	119 (96 to 130)	80 (65 to123)	107 (99 to 126)
Respiratory rate	26 (20 to 33)	33 (27 to 39)	20 (15 to24)	24 (20 to28)
**Organ dysfunction at enrollment**				
PaO2/FiO2 ratio	128 (105 to 209)	126 (94 to 198)	127 (72 to 211)	145 (81 to 223)
Creatinine (mg/dL)	1.7 (0.8 to 2.9)	1.0 (0.8 to 1.8)	1.2 (1.0 to 1.7)	0.9 (0.8 to 1.4)
Vasopressors	32%	56%	19%	5%
Bilirubin (mg/dL)	0.5 (0.4 to 0.8)	0.9 (0.4 to 1.8)	0.6 (0.5 to 1.6)	0.6 (0.4 to 1.1)
Platelets (10^3^/μL)	176 (61 to 304)	183 (107 to 266)	186 (101 to242)	145 (114 to 242)

Median (interquartile range) unless otherwise noted.

APACHE II, Acute Physiology and Chronic Health Evaluation II; BP, Blood pressure; DEX, dexmedetomidine; FiO2, fraction of inspired oxygen; IQCODE, Informant Questionnaire on Cognitive Decline in the Elderly; LZ, lorazepam; PaO2, partial pressure of arterial oxygen; RASS, Richmond Agitation-Sedation Scale; SIRS, Systemic Inflammatory Response Syndrome; SOFA, Sequential Organ Failure Assessment.

### Major clinical outcomes and mortality

**Figure 1:**
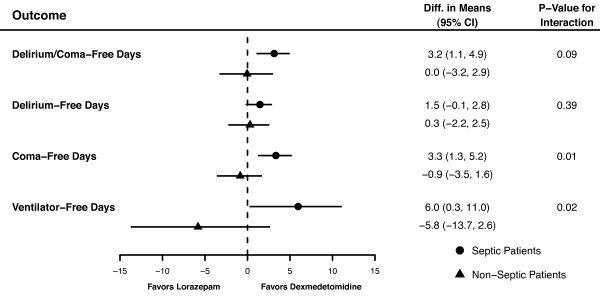
**Forest plot demonstrating interactions between sepsis and the effect of sedative group on delirium/coma-free days, delirium-free days, coma-free days, and ventilator-free days**. For each outcome, the adjusted difference in the means between the dexmedetomidine group and lorazepam group is presented, first for the septic patients (heavy circle) and then for the non-septic patients (heavy triangle), along with 95% confidence intervals (CI) for the difference. Differences, CIs and *P *values were calculated using bootstrap multiple linear regression, adjusting for age, the acute physiology component of the Acute Physiology and Chronic Health Evaluation (APACHE) II score at enrollment, administration of drotrecogin alfa (activated), treatment group, sepsis, and treatment group by sepsis interaction. If the difference in means is greater than 0, it reflects an improved outcome with dexmedetomidine; if less than 0, then patients on lorazepam had a better outcome. We considered a *P *value for interaction less than 0.15 to indicate that the effect of sedative group on the outcome in question was different for septic patients than for non-septic patients. A *P *value for interaction of 0.15 or more, alternatively, indicated that the effect of sedation group on outcomes was the same for all patients, regardless of sepsis.

**Figure 2:**
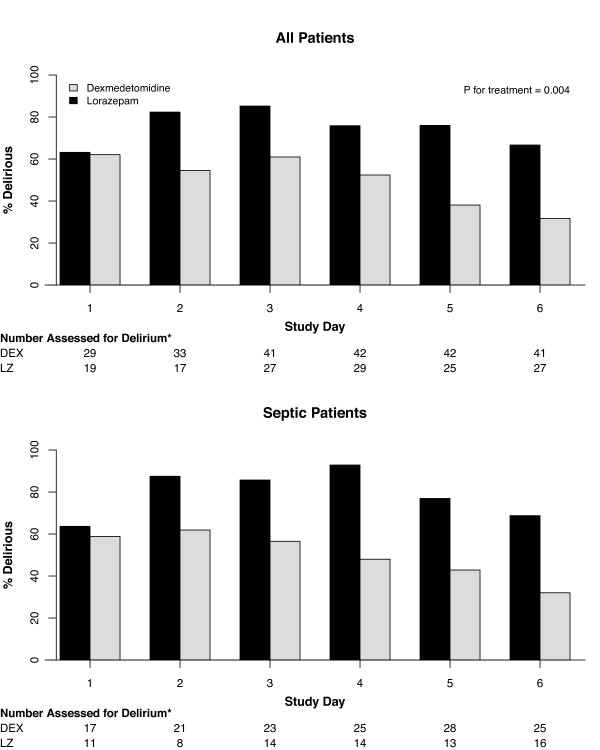
**Prevalence of delirium while on study drug**. The top panel demonstrates that, among all patients, those sedated with dexmedetomidine (DEX) had a 70% lower likelihood of having delirium on any given day compared with patients sedated with lorazepam (LZ). Sepsis did not modify this relation (adjusted *P *for interaction = 0.94), meaning that dexmedetomidine reduced the risk of developing delirium whether patients had sepsis (lower panel) or not. * Number of patients assessed denotes the number of patients who were alive, in the ICU, and not comatose (Richmond Agitation-Sedation Scale (RASS)-3 or lighter) and are therefore assessable for delirium. Percentages of patients alive and without coma, but with delirium, are represented with black bars if on lorazepam and gray bars if on dexmedetomidine.

Septic patients sedated with DEX had a mean (95% CI) of 3.2 (1.1 to 4.9) more delirium/coma-free days, 1.5 (-0.1 to 2.8) more delirium-free days, and 6 (0.3 to 11.0) more ventilator-free days than patients receiving LZ, after adjusting for relevant covariates. However, no substantial difference was seen in these outcomes between non-septic patients treated with DEX and LZ (Figure 1 and Table 2). Sedation with DEX had a greater impact on patients with sepsis compared with those without sepsis for delirium/coma-free days (*P *for interaction = 0.09) and for ventilator-free days (*P *for interaction = 0.02; Figure 1). Alternatively, the effect of DEX vs LZ sedation on the probability of being delirious was the same for septic and non-septic patients (*P *for interaction = 0.94); among all patients (regardless of sepsis), DEX-treated patients had 70% lower odds, compared with LZ-treated patients, of being delirious on any given day (odds ratio (OR) = 0.3, 95% CI = 0.1 to 0.7; Figure 2). Amongst the four CAM-ICU features, the beneficial effects of DEX (vs LZ) on delirium outcomes were driven by lower odds of development of inattention (CAM-ICU Feature 2; OR = 0.3, 95% CI = 0.1 to 0.7; *P *= 0.005) and disorganized thinking (CAM-ICU Feature 3; OR = 0.2, 95% CI = 0.1 to 0.5; *P *< 0.001) (i.e. features associated with content of arousal), and not as much by level of arousal.

**Table 2:** Outcomes of patients with and without sepsis*

	Patients with sepsis			Patients without sepsis		
		
Outcome variable	DEX (n = 31)	LZ (n = 32)	Adjusted *P *value**	DEX (n = 20)	LZ (n = 19)	Adjusted *P *value**
**Duration of brain organ dysfunction**						
Delirium/coma-free days**	6.1 (4.3)	2.9 (3.2)	0.005	6 (4.7)	5.5 (3.6)	0.97
Delirium-free days^†^	8.1 (3.1)	6.7(2.9)	0.06	8.1 (3.5)	7.9 (2.8)	0.80
Coma-free days^ǂ^	9.4 (2.9)	5.9 (4.2)	<0.001	8.9 (4)	8.8 (2.6)	1
**Other clinical outcomes**						
MV-free days^‡^	15.2 (10.6)	10.1 (10.3)	0.03	12.8 (11.5)	17.2 (10)	0.15
ICU days	13.4 (15.1)	12.2 (9.8)	0.81	14.9 (16.5)	10.4 (8.9)	0.28
28-day mortality	16%	41%	0.03	19%	5%	0.21

Mean (standard deviation) unless otherwise noted.

DEX, dexmedetomidine; LZ, lorazepam; MV, mechanical ventilation.

* Adjusted *P *value obtained from the bootstrap multiple linear regression that calculated a difference in mean for each outcome between the two treatment groups, adjusting for age, severity of illness, use of drotrecogin alfa (activated) within 48 hours of enrollment, sepsis, treatment group, and a treatment group by sepsis interaction.

**Indicates the number of days alive without delirium or coma from study day 1 to 12.

†Indicates the number of days alive without delirium from study day 1 to 12.

ǂIndicates the number of days alive without coma from study day 1 to 12.

‡Indicates the number of days alive breathing without assistance of the ventilator from study day 1 to 28.

**Figure 3:**
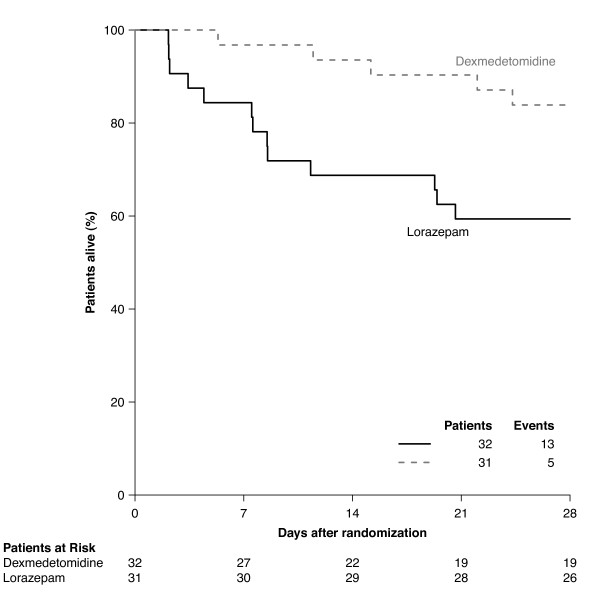
**Kaplan-Meier curve showing probability of survival during the first 28 days according to treatment group, among patients with sepsis**. Dexmedetomidine decreased the probability of dying within 28 days by 70%; this beneficial effect was not seen in patients who were not septic (*P *value for interaction = 0.11 implying an interaction between sepsis and the treatment groups).

Septic patients sedated with DEX additionally had a lower risk of death at 28 days as compared with those sedated with LZ (hazard ratio (HR) = 0.3, 95% CI = 0.1 to 0.9; Figure 3); however, this beneficial effect was not seen in non-septic patients (HR = 4.0, 95% CI = 0.4 to 35.5; *P *for interaction = 0.11). The proportional hazards assumption for time to death within 28 days was validated graphically and via examining model residuals [50].

### Efficacy of sedation

Among the septic patients, those sedated with DEX achieved sedation within one point of their ordered RASS target more often than those sedated with LZ (accurately sedated on 67% of days (50 to 83%) vs 52% of days (0 to 67%), *P *= 0.01); however, efficacy of sedation among the non-septic patients was similar for both treatment groups (67% of days (50 to 86%) vs 60% of days (27 to 75%), *P *= 0.27). Median (interquartile range) DEX dose was 0.8 mcg/kg/hour (0.3 to 1.1) and LZ dose was 3.6 mg/hr (2.2 to 7.1) in the septic patients. In the non-septic group, median infusion rate were 0.6 mcg/kg/hr for DEX and 2.7 mg/hr for LZ. Septic patients sedated with DEX received more fentanyl per day (1,114 mcg/day (212 to 2997) vs 117 (0 to 1460), *P *= 0.01) than septic patients sedated with LZ, while fentanyl use was similar in the non-septic DEX and LZ groups (520 mcg/day (133 to 1778) vs 262 (10 to 775), *P *= 0.20).

### Safety evaluation

Incidence of hypotension, vasopressor use and cardiac arrhythmias monitored during the study are shown in Table 3. There were no differences in cardiac, hepatic, renal, and endocrine functional, and injury parameters between the DEX and LZ groups, regardless of sepsis at enrollment (all *P *> 0.10). Development of new secondary infections beyond the first 48 hours after enrollment was similar in the originally non-septic group in the DEX and LZ study arms (17% vs 15%).

**Table 3:** Hemodynamic parameters in patients with and without sepsis

	Patients with sepsis			Patients without sepsis		
		
Hemodynamic variable*	DEX (n = 31)	LZ (n = 31)	*P *value	DEX (n = 20)	LZ (n = 19)	*P *value
Number of days on vasoactive drugs	1 (1)	2 (2)	0.08	1.5 (2.2)	0.3 (0.9)	0.08
Average daily number of vasoactive drugs	1.1 (0.2)	1.6 (0.5)	0.004	1.6 (0.9)	1 (0)	0.2
Ever vasoactive drugs increased	26%	47%	0.08	33%	16%	0.2
Sinus bradycardia (<60 beats/min)	13%	6%	0.4	24%	0%	0.02
Sinus tachycardia (>100 beats/min)	81%	84%	0.7	52%	53%	1

Mean (standard deviation) unless otherwise noted.

DEX, dexmedetomidine; LZ, lorazepam.

*Measured during 120-hour study drug protocol, except for sinus bradycardia and sinus tachycardia, which are measured during entire study.

## Discussion

This subgroup analysis presents data indicating that the choice of a sedative may be important for sepsis patients in determining clinical outcome. Septic patients treated with DEX had shorter duration of acute brain dysfunction (delirium and coma), lower daily probability of delirium, shorter time on the ventilator, and improved 28-day survival as compared with septic patients treated with LZ. Our results further suggest that sedation regimens incorporating DEX have a greater impact on these important outcomes in patients with sepsis than in patients without sepsis. These findings suggest that choice of sedative is vitally important in the vulnerable septic patient population and, along with other strategies [51], needs to be addressed at the time sedative regimens are initiated for MV.

Our findings could be the result of either a beneficial effect of DEX in the setting of sepsis, a deleterious effect of LZ in this setting, or both [27]. Benzodiazepines inhibit macrophage function [31,32], whereas α_2 _adrenoceptor agonists appear to promote macrophage phagocytosis and bactericidal killing [34-36]. Given the crucial role of macrophage function in mucosal immunity and clearance of bacteria, the opposing effects of these sedatives on macrophages may, at least in part, explain our findings herein. These alternate effects on macrophage function are also consistent with the reduced number of secondary infections experienced in DEX-sedated (vs midazolam-sedated) patients in a secondary analysis from the recent SEDCOM trial [23], although a cursory look at our own data showed no differences in new infections.

Nonetheless the mortality benefit that was provided by DEX over LZ in our patients with sepsis may be due to several factors. These include differences in the effects of these sedative regimens on both innate immunity and inflammation [27] and also on the anti-apoptotic role of DEX [40,42] that may mitigate the deleterious effect of apoptosis in the pathogenesis of sepsis [43]. Indeed, we have recently observed that DEX reduces the burden of apoptosis from severe sepsis to a greater degree than midazolam in the cecal ligation and puncture model [40]. Furthermore, the anti-inflammatory effects of DEX may have also contributed to both the reduction in the risk of delirium and the shorter duration of brain dysfunction because inflammation likely plays an important role in the pathophysiology of ICU delirium [12,13]. The benefits provided by DEX may also be attributed to consequences of the quality of sedation. DEX sedation is more akin to non-rapid eye movement sleep, than is sedation with benzodiazepines [22,24]; thus, it is possible that improved sleep in critically ill patients could have contributed to improved outcomes given the relation between sleep with immunity and delirium [12,25,26]. Sleep deprivation has been associated with higher levels of both pro- and anti-inflammatory cytokines, decreased glucose tolerance and increased insulin resistance and activation of the hypothalamic-pituitary axis [26]; all of these can contribute to worse clinical outcomes [26,52]. Previous polysomnographic studies have revealed that intensive care patients sleep for less than two hours in a 24-hour period; thus, prolonged stays in intensive care may result in a huge sleep debt with all the attendant complications of sleep deprivation [25,26]. The putative contribution of the more natural sleep-enhancing properties of DEX [22,24] to the observed outcome benefits in septic patients requires further investigation.

We did not observe any adverse events in the septic DEX group (with the possible exception of bradycardia), and there were no differences in liver, renal, cardiac, or endocrine safety outcomes (e.g., cortisol levels) in septic patients treated with DEX vs LZ, attesting to its safety in critically ill septic patients. DEX has been reported to cause hypotension and bradycardia in patients, due to the inhibition of central norepinephrine release, peripheral vasodilation and a vagomimetic action [22]. Although this may be concerning in septic patients who are at risk for the development of shock, we observed no difference in the incidence of hypotension between treatment groups. In fact, DEX-treated patients required fewer daily vasopressors and had trends towards shorter duration of hypotension that may reflect improvement in sepsis severity due to the putative effects of DEX on inflammation and immunity. This reduction in vasopressor use in the septic patients is corroborated by a decrease in hypotension seen in animals receiving DEX during septic shock [38,39] and reduced patient epinephrine requirements in DEX-treated patients following cardiac surgery [53]. In the animal studies, the improved hemodynamic stability correlated with reduced inflammation following DEX administration [38-40]. Indeed in two recent studies, DEX sedation has been associated with a reduction in pro-inflammatory cytokines in patients with sepsis relative to midazolam [54] and propofol [55]. It is plausible that hemodynamic-stabilizing and anti-inflammatory effects of DEX are linked by central sympatholysis [27,38,39]; although appearing counter-intuitive, we consider that a reduction in pro-inflammatory cytokines would outweigh any direct hypotensive effect of DEX [27,38,39], the net effect being improved hemodynamic stability.

Although fentanyl doses were significantly greater in septic DEX-treated patients than in LZ-treated patients -- likely because supplemental analgosedation may be needed to achieve heavy sedation for a DEX-treated patient -- it is unlikely that the benefits observed in the DEX group were attributable to the use of fentanyl. Indeed, available evidence indicates that opioids have immunosuppressive effects and are capable of increasing mortality in animal models of infection [27,56]. Additionally, fentanyl may contribute to delirium [6]. Thus, we would expect the increased opioid use in the DEX group to have reduced rather than promoted the observed benefits.

Interestingly, although we observed significant benefits of α_2 _adrenoceptor agonist based sedation compared with GABAergic sedation in septic patients, we did not observe all the benefit in the non-septic group. DEX-treated patients did have lower odds of development of delirium, whether septic or non septic; however, the improvements in duration of brain dysfunction were predominantly seen in the septic patients on DEX. This may be because the non-septic group was smaller than the septic group and thus had limited statistical power to identify any beneficial or detrimental effect of either treatment. Additionally differences in pathogenesis of delirium may account for the greater benefit seen in septic patients. Furthermore septic shock is associated with neuronal apoptosis in the brain, including the locus ceruleus [57], where there is an abundance of α_2 _adrenoceptors. Given that DEX prevents central neuroapoptosis via activation of α_2 _adrenoceptors [42], these neuroprotective effects may have contributed to the benefits observed in the septic group to a greater extent than in the non-septic group.

There are several limitations to this investigation. First, we categorized patients as septic and non-septic based on the presence of at least two SIRS criteria and suspected infection, in accordance with the consensus definition [52]. As in clinical practice, these determinations were not always supported by microbiological evidence. However, a certified critical care physician confirmed all suspected cases of sepsis to ensure that postoperative patients on prophylactic antibiotics were not misclassified as septic. Future prospective studies should include referral to a clinical evaluation committee to confirm the diagnosis of sepsis and appropriateness of other therapeutic interventions designed to survive sepsis. Patients were classified as septic if they met criteria from admission up to 48 hours after enrollment, to avoid potential for misclassification. However previous analysis of these data [58], where patients were classified by pre-randomization admission diagnosis of sepsis, found similar results to those presented herein, strengthening our findings. Second, this is a subgroup analysis of a larger study, and the study was not powered to specifically examine interactions. Our data are therefore vulnerable to type II error, and we advise cautious interpretation of these preliminary findings [59-61]. Interestingly, differences in the magnitude of a treatment effect based on subgroup analyses are commonplace, however, as further evidence accumulates qualitative differences (differences in the direction of treatment effect) are rarely found [62-64]. Third, the subset population of septic individuals in the MENDS trial may not be generalizable to the entire septic population because of certain exclusion criteria, including severe liver failure, alcohol abuse, and ongoing cardiac ischemia. Fourth, randomization was not specifically applied to the septic and non-septic cohort and hence demographic imbalances, common in subgroup analyses, could have occurred. Fortunately, the DEX and LZ groups were balanced for several important criteria, including severity of illness and organ failure scores (Table 1). However, some imbalances did exist; for example, more non-septic patients randomized to DEX were admitted to the medical ICU, which often have higher mortality than surgical ICUs due to associated comorbidities. We were unable to assess whether this difference had a role in the non-significant trends towards lower survival in the DEX non-septic group as compared with the LZ non-septic patients. We did, however, try to account for potential confounding by including important clinical covariates in our model (including age, severity of illness according to the acute physiology component of the APACHE II score at enrollment, and use of drotrecogin alfa (activated) within 48 hours of enrollment). Finally, the MENDS study was designed to compare DEX with the current recommended sedative, LZ. Further studies are required to understand whether DEX is similarly superior to other benzodiazepine and non-benzodiazepine agents, such as propofol, that also act via the GABA_A _receptor. Indeed, LZ is a significant risk factor for delirium [18] and may have exaggerated any perceived benefit from DEX; it is therefore important that future studies concentrate on alternate agents. These studies should also focus on long-term outcomes such as 90-day mortality to ensure a persistent survival benefit. Thus, these results must be confirmed in an adequately powered prospective phase IIb and phase III studies before widespread changes are made to clinical practice.

## Conclusions

In this *a priori*-identified subgroup analysis, sedation with DEX reduced the duration of brain organ dysfunction, lowered the probability of delirium, increased time-off mechanical ventilation, and reduced 28-day mortality as compared with LZ in septic patients; the benefit of DEX sedation was greater for septic patients than for non-septic patients in terms of duration of acute brain dysfunction (delirium or coma), time on mechanical ventilation, and mortality. Prospective multicenter, randomized controlled trials are needed to confirm these results and examine the mechanisms underlying the effect of DEX on outcomes, including mortality, in sepsis.

## Key messages

• In this *a priori *designed subgroup analysis of the MENDS study, septic patients receiving DEX had more days free of brain dysfunction and MV and were less likely to die than those that received a LZ-based sedation regimen. Patients on DEX had lower odds of developing delirium whether septic or non-septic as compared with those on LZ.

• The majority of benefits conferred by DEX sedation were more prominent in septic patients than in non-septic patients.

• Further prospective clinical and preclinical study is warranted into the potential benefits of sedation with drugs targeting the α_2 _adrenoceptor rather than the GABA_A _receptor.

## Abbreviations

APACHE: Acute Physiology and Chronic Health Evaluation; CAM-ICU: Confusion Assessment Method for the ICU; CI: confidence interval; DEX: dexmedetomidine; HR: hazard ratio; LZ: lorazepam; MV: mechanical ventilation; OR: odds ratio; RASS: Richmond Agitation-Sedation Scale; SIRS: systemic inflammatory response syndrome.

## Competing interests

PPP, DLH, MM and TDG have received research grants or honoraria from Hospira Inc. EWE has received research grants and honoraria from Hospira, Inc, Pfizer, and Eli Lilly, and a research grant from Aspect Medical Systems. All other authors report that they have no competing interests.

## Authors' contributions

RDS developed the hypothesis with PPP, MM and EWE. All authors were involved in the study design and interpretation. The analysis was performed by PPP, TDG, SM, AKS, JLT and EWE. All authors contributed to data interpretation. Primary responsibility for drafting the manuscript lay with PPP and RDS who contributed equally to the paper.
